# The role of HnrnpF/H as a driver of oligoteratozoospermia

**DOI:** 10.1016/j.isci.2024.110198

**Published:** 2024-06-06

**Authors:** Jacob K. Netherton, Rachel A. Ogle, Benjamin R. Robinson, Mark Molloy, Christoph Krisp, Tony Velkov, Franca Casagranda, Nicole Dominado, Ana Izabel Silva Balbin Villaverde, Xu Dong Zhang, Gary R. Hime, Mark A. Baker

**Affiliations:** 1 School of Biomedical Sciences and Pharmacy, Faculty of Medicine and Health, University of Newcastle, Callaghan, NSW 2308, Australia; 2 Australian Proteome Analysis Facility, Department of Biomolecular Sciences, Macquarie University, NSW 2109 Australia; 3 Biomedicine Discovery Institute, Infection & Immunity Program and Department of Microbiology, Monash University, Clayton, VIC 3168, Australia; 4 Department of Anatomy and Physiology, University of Melbourne, Parkville, VIC 3010, Australia; 5 Institute of Biological and Natural Sciences, Federal University of Triângulo Mineiro, Uberaba, Minas Gerais, Brazil

**Keywords:** reproductive medicine, cell biology

## Abstract

Male subfertility or infertility is a common condition often characterized by men producing a low number of sperm with poor quality. To gain insight into this condition, we performed a quantitative proteomic analysis of semen samples obtained from infertile and fertile men. At least 6 proteins showed significant differences in regulation of alternatively spliced isoforms. To investigate this link between aberrant alternative splicing and production of poor-quality spermatozoa, we overexpressed the hnrnpH/F-orthologue Glorund (Glo) in *Drosophila,* which was also found to be abundant in poor quality human sperm. Transgenic animals produced low numbers of morphologically defective spermatozoa and aberrant formation of the “dense body,” an organelle akin to the mammalian manchette. Furthermore, fertility trials demonstrated that transgenic flies were either completely infertile or highly subfertile. These findings suggest that dysregulation of hnrnpH/F is likely to result in the production of low-quality semen, leading to subfertility or infertility in men.

## Highlights

• The RNA splicing factor HnrnpF/H is more abundant in infertile human spermatozoa
• Infertile human spermatozoa possess aberrant alternative splicing events
• Overexpressing the *Drosophila* HnrnpF/h orthologue Glo causes poor quality sperm
• Overexpression of Glo causes infertility or severe male subfertility in *Drosophila*

## Graphical abstract

**Figure:**
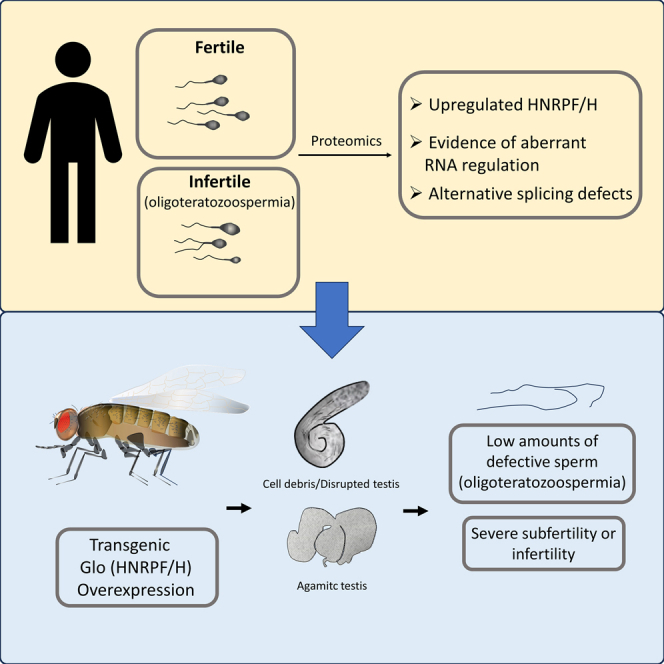

## Introduction

Male sub- or infertility is a medical condition that affects approximately one out of every 20 men in the western world.^1^^,^^2^ In the United States, 28% of couples seeking assisted conception (AC) services do so primarily due to male-factor.^3^ Clinical examination of the semen from infertile men reveals three significant factors that impede reproductive success: reduced motility (asthenozoospermia), low sperm count (oligozoospermia), and abnormal morphology (teratozoospermia). Clinically, a combination of these phenotypes is typically seen, such that in 50% of the cases men present with at least two of these phenotypes. Of the remaining infertile men, 20% have no sperm in their semen (azoospermia), while 12% display normal sperm parameters (4).

Determining the precise quantity of normal, motile spermatozoa necessary for male fertility has posed challenges.^4^ A semen analysis involving 765 infertile couples (no pregnancy after 12 months) and 696 fertile couples (conceived within the previous 2 years)^5^ showed fertile men produced over 48 × 10^6^ sperm/mL with >63% motility and >12% normal morphology.^5^ In contrast, those men from infertile couples had an average sperm concentration lower than 13 × 10^6^ sperm/mL, motility <32% and normal morphology forms of <9%.^5^ Thus, in general, it is understood that the greater concentration of morphologically normal, motile sperm present in the ejaculate, the better fecundity a man will have. In agreement, the World Health Organization commissioned a study consisting of 4500 men and examined their time-to-pregnancy within one year.^6^ Their findings show that men who produced sperm concentrations >15 × 10^6^ sperm cells/mL with >32% for progressive motility and >4% normal forms^6^ were in the 5^th^ centile to conceive.^6^

Considering the critical role of normal sperm production in male fertility, our research is primarily focused on unraveling the molecular foundations of male-factor infertility. Several lines of evidence suggest that one contributing factor to this condition is aberrant RNA regulation, specifically alternative splicing. For example, the RNA binding protein ADAD2 regulates alternative splicing of RNA species from the pachytene stage onward. The elimination of this gene results in oligoasthenoteratozoospermia and subsequent sterility.^7^ Similarly, deletion of brother of the regulator of imprinted sites (BORIS), which regulates the short RNA isoform of cerebroside sulfotransferase (CST), produces mice with the same phenotype. In addition, knockout of the RNA binding proteins Sam68^8^ MRG15,^9^ ptbp2, ^10^ and RBM5^11^ all lead to male infertility.

Further to this, there is a growing body of evidence to suggest changes within alternative splicing regulators are physiologically relevant to male-infertility. Alternative splicing is regulated by two groups of proteins, known as splicing enhancers or silencers. At least 12 splicing enhancers have been characterised^12^ and these proteins position themselves close to the intron-exon boundary of pre-mRNA. They “recruit” the spliceosome complex, which then cuts the appropriate nucleotides within the pre-mRNA to remove introns.^13^ On the other hand, splicing silencers inhibit alternative splicing in different ways, such as: (i) binding to splicing enhancers directly to displace/block their ability to bind pre-mRNA; (ii) binding to spliceosome subunits to block the formation of the spliceosome, and (iii) binding and folding pre-mRNA into a loop, thereby inhibiting splicing.^13^ Over 30 different types of splicing silencers have been characterised,^14^ many of which are part of the heterogeneous nuclear ribonucleoprotein (Hnrnp) family. Loss of a single copy of the testis-specific RNA regulator heterologous ribonucleoprotein G-T (hnRNP G-T)^15^ or the Y chromosome deletions, which cover a number of RNA binding proteins including RBM and DAZ,^16^ result in either no or low amount of poor sperm being produced, with evidence of sub and male infertility. In addition, conditional deletion of the heterogeneous nuclear ribonuclear protein hnRNPH1 within the germ line, leads to both male and female infertility, with aberrant mRNA splicing seen in the male germs cells.^17^

A potential reason as to why the testis are so exquisitely sensitive to aberrant RNA regulation, is their unique and high usage of alternative splicing.^18^ Not only is the testis considered to undertake more alternative splicing events besides the brain,^18^ several testis-specific splicing events such as alternative 5′ or 3′ splice sites and intron retention^18^ events have been reported.

In this study, we performed a quantitative proteomic analysis of semen samples from individuals diagnosed with oligoteratozoospermia (OT) to gain a deeper understanding of the underlying causes of this condition. While several proteins were found to change in terms of abundance, our data point to a strong link between RNA dis-regulation and OT. RNA metabolism was shown to be an enriched pathway with several alternative splicing regulators found to be more abundant within the infertile samples. Furthermore, for the first time, we show poor quality sperm samples possess different abundance of alternatively spliced protein isoforms, suggesting this could be a possible cause. As such, in proof-of-concept, overexpression of the hnRNPH orthologue (an alternative splicing regulator found to be more abundant within infertile samples), “Glorund” (Glo) in *Drosophila,* lead to the condition of OT or oligozoospermia in various strains. Furthermore, fertility trials demonstrated the flies were either severely subfertile or infertile. As such, our analysis indicates that aberrant regulation of RNA during spermatogenesis could be a primary cause of a lack of poor quality spermatozoa.

## Results

In order to gain a mechanistic insight into male-infertility, we performed a quantitative proteomic analysis using semen samples obtained from fertile men (time-to-pregnancy less than one year) against infertile men (no pregnancy within one year of trying and no obvious female factor). On average, the fertile cohort produced sperm concentrations of 181 × 10^6^ sperm/mL (range: 72–340 × 10^6^ cells/mL) with 36% normal morphology (range: 24–50%) and 71% average motility (range: 53–88%). The values for cell concentration and morphology places these men in the 75^th^–90^th^ centile category for fertility within one year, and for motility within the 50 ^th^ −75^th^ centile.^5^ In contrast, infertile men produced low numbers of spermatozoa, with an average of 25 × 10^6^ sperm/mL (range: 5–45 × 10^6^ cells/mL), with 5.6% normal morphology (range 4–8%) and 44.5% motility (range 37–64%); consistent with an OT phenotype. On average, these infertile samples were in the 10^th^ percentile of men able to conceive within one year. The individual semen parameters of all the men used in this study are given in Table SI. Table 1 lists the average of semen parameters of each individual and the centile range they fall in.

**Table 1:** Average semen quality (mean ± SD) and WHO classification (centile category for time-to-pregnancy) of the sperm samples selected for the proteomic study

	Average [conc]	Average normal forms (%)	Average motility (%)	WHO centile [conc]	WHO centile [morph]	WHO centile [mot]
Fertile donors	239.1 ± 81.6^a^	36.2 ± 9.8^a^	71.2 ± 11^a^	75–90	75–90	50–75
Infertile donors	24.8 ± 14.1^b^	5.6 ± 1.5^b^	44.5 ± 7.1^b^	10	10	10–25

a Typical semen analysis of men able to conceive within one year as defined by Guzick et al.^5^.

b Typical semen analysis of men unable to conceive within one year as defined by Guzick et al.^5^.

**Figure 1:**
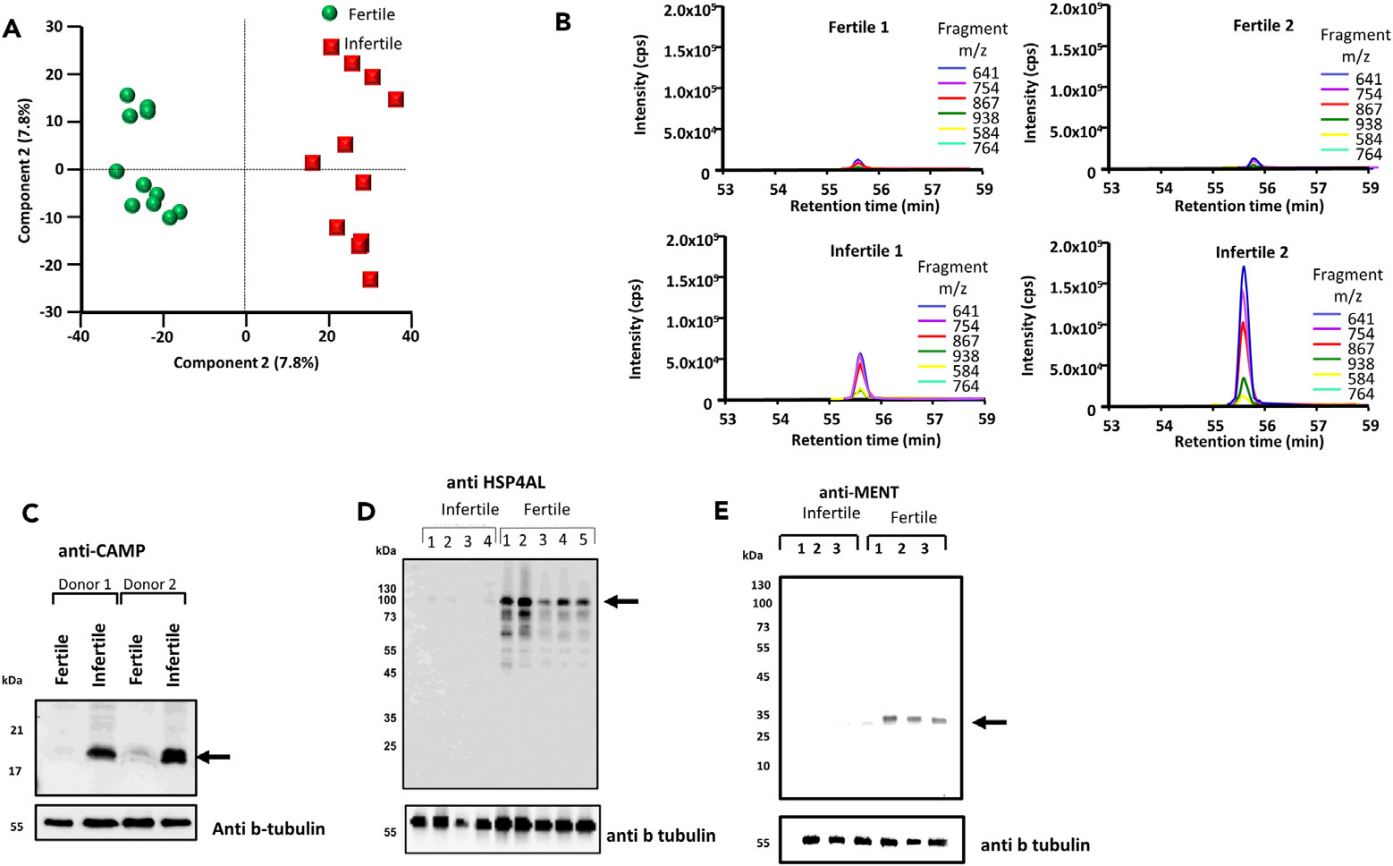
Aberrant protein changes and alternative splicing in men with poor semen quality Human spermatozoa were collected from both fertile and infertile men. After lysis, the samples were digested and run through quantitative mass spectrometry. (A) Principal component analysis showing separation of both fertile (green) and infertile (red) men based on the protein changes in the proteomic analysis. (B) Extracted ion chromatogram of peptide derived from CAMP from 2 fertile (top) and 2 infertile (bottom) samples (C–E) sperm lysates from fertile and infertile men were lysed and run into SDS page. The samples were then probed for using the (C) anti-CAMP, (D) anti-HSP4AL, and (E) anti-MENT antibody. The membrane was then stripped and re-probed with anti-beta tubulin as a loading control. The position of the molecular mass markers are shown on the left hand side.

Using a label-free quantitative proteomic approach (SWATH), we found 369 protein changes (from a total of 1417 identifications) that were significantly different (*p* < 0.05) with at least a 2-fold change in protein abundance between the fertile and infertile groups (Table S2). Principal component analysis showed that the fertile (Figure 1A, green) and infertile (Figure 1A, red) groups separately clustered, suggesting each of the groups had unique protein differences. An example of one change is shown in Figure 1B, where eluting fragment peptides derived from cathelicidin antimicrobial peptide (CAMP) is shown from two fertile patients (top panels) and two infertile patients (bottom panel). Here, it is clear that CAMP is more abundant within the infertile samples. To confirm this, we selected two random infertile and fertile samples, then probed them with the anti-CAMP antibody. Consistent with the protein analysis, CAMP was found to be more abundant within the fertile patient co-hort. In addition, we also probed different fertile and infertile samples with anti-HSP4AL (Figure 1D) and anti MENT (Figure 1E). Both proteins were shown to be more abundant in the fertile samples, which is in agreement with the proteomic analysis.

**Figure 2:**
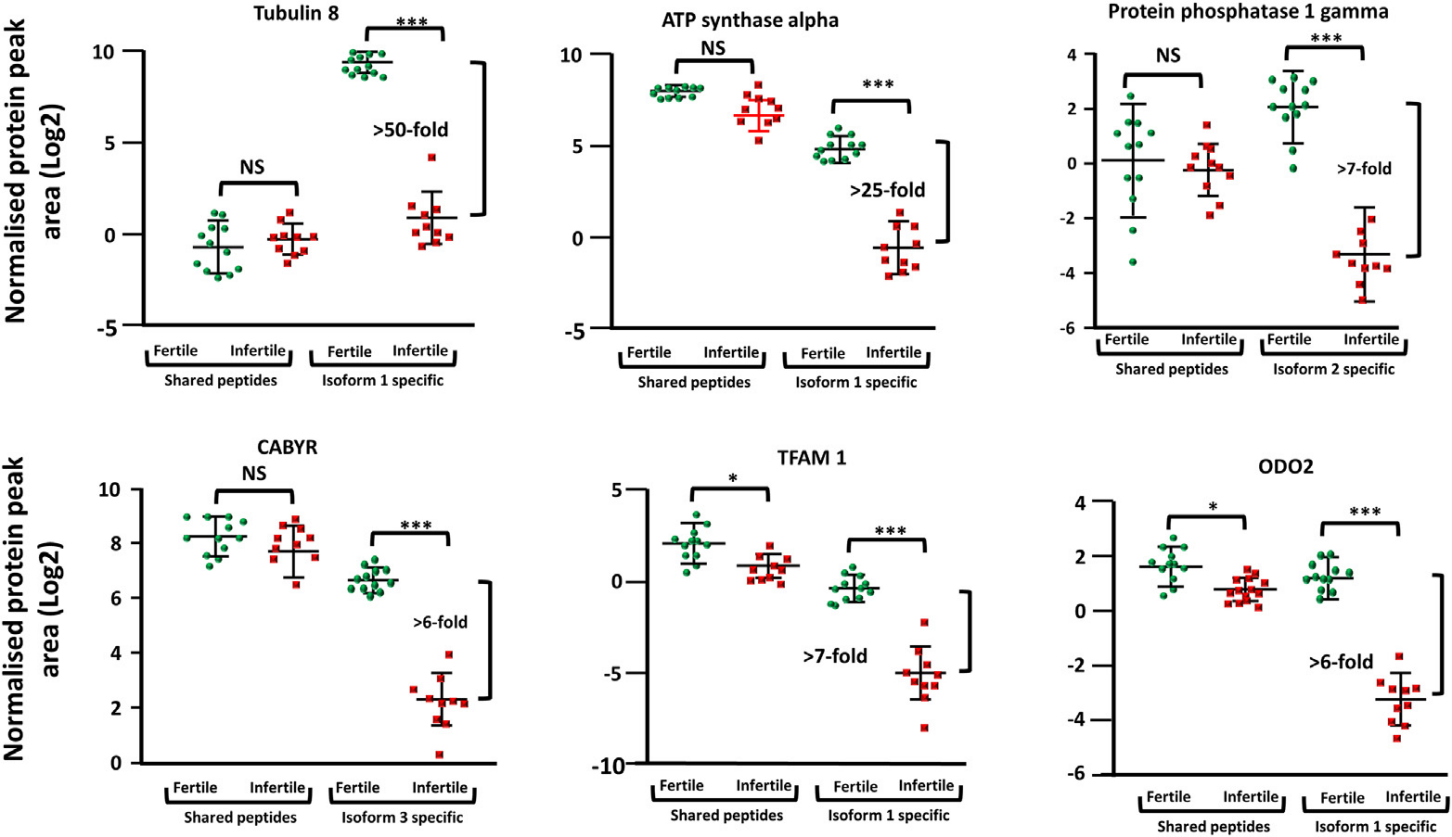
Aberrant alternative splicing is a hallmark of poor sperm quality, Peptides either common to, or specific for one of the protein isoforms for each sample were extracted and the area of elution for each fragment ion was concatenated. Following normalization, the difference in peptide expression for both the common peptides and the isoform specific peptides are shown. Interquartile ranges are shown. ∗ = *p* < 0.05, ∗∗∗ = *p* < 0.001 (Student's t test), NS = not significant.

Having confirmed the validity of our dataset, we next undertook a bioinformatic analysis of differentially expressed proteins to gain insight into which pathways may be affected. It should be noted that gene ontology analysis needs to be considered carefully when dealing with spermatozoa as the cell is so highly differentiated, that, for example, many proteins considered “cytosolic” in somatic cells, are actually present on the sperm flagella.^19^^,^^20^^,^^21^ Despite this, at the sub-cellular level, mitochondrial proteins were the most significantly regulated (Table S3). A perusal of this list showed enrichment of Hsp70, Hsp60, and Hsp10, which act on misfolded proteins during the formation of a poor-quality sperm. Changes to proteins in the nucleosome were ranked second in terms of their significance, which like the mitochondria was not unexpected as the head shape of infertile spermatozoa is often deformed. From a biological process point of view, the most significantly enriched category were proteins involved in RNA binding, which among others included several exon skipping regulators (e.g., hnRNP F/H, hnRNP A3, and hnRNP C), histone 3, and matrin 3 (a nuclear matrix protein involved in various cellular processes, including RNA splicing, transcriptional regulation, and DNA repair), together with several proteins that make up the core of the spliceosome (e.g., snRNP D1 and D3). While changes to both mitochondria and nucleus likely reflect the morphological defects seen in human spermatozoa, changes in RNA regulation suggested that this pathway may be at the etiology of poor sperm formation. With that in mind, we next wanted to demonstrate if there was any evidence that alternative splicing could be found in the sperm samples. For this cause, we developed a strategy (R script) which found proteins that had peptides which were common between protein isoforms, and (other peptides) unique for specific protein isoforms. We then looked to see if we could see abundance difference between the common peptides and the specific isoform proteins which would not be detected using a common proteomics pipeline. After protein area normalization, two-sample Student’s t tests were performed and the protein list was filtered for isoform specific protein groups that showed differences in the expression levels between fertile and infertile samples (*p* < 0.01). Applying the filters mentioned previously, six proteins were identified with discrepancies in isoform specific expression (Figure 2).

The largest expression differences were observed for tubulin alpha 8 (TUBA8) isoforms. Peptides shared between isoform 1 and 2 did not show differences in expression between fertile and infertile samples (1.5-fold higher in infertile men group). However, the isoform 1 specific peptide (AVMIDLEPTVVDEVR) is more than 50-fold higher in the fertile men group (*p* = 2.5 e^−13^, Figure 2) suggesting aberrant alternative splicing of this protein. The peptidyl-prolyl *cis*-trans isomerase G (PP1G) also showed differences in isoform expression. Peptides shared between PPIG isoforms had no expression change between the two groups, however, the peptide specific for isoform 2 (VASGLNPSIQK) was 16-fold lower in the infertile men group (*p* = 2.1 e^−7^, Figure 2). Similarly, a 6-fold change in expression was found in the calcium-binding tyrosine phosphorylation-regulated protein (CABYR), isoform 3 infertile men (Figure 2). Isoform specific changes were also observed for the mitochondrial transcription factor A (TFAM, isoform 1, 7-fold lower in infertile men group, *p* = 1.3 e^−5^), mitochondrial ATP synthase subunit alpha (ATPA, isoform 1, 25-fold lower in infertile group, *p* = 4.5 e^−11^) and mitochondrial dihydrolipoyllysine-residue succinyltransferase component of 2-oxoglutarate dehydrogenase complex (ODO2, 6-fold lower in infertile men, *p* = 3.3 e^−7^, Figure 2). These data suggest that changes in alternative splicing were a hallmark of OT.

To understand how aberrant alternative splicing may have occurred, we re-investigated the proteomic lists looking for proteins involved in mRNA alternative splicing with a significant change in abundance (*p* < 0.05, >2-fold difference). Candidate proteins that could facilitate production of different protein isoforms were identified, included hnRNP H/F (2.6-fold higher within infertile samples) and hnRNP C (6.9-fold higher within infertile samples).

### Overexpression of hnRNP H/F homologue, Glo, in *Drosophila* germ cells, results in loss of sperm production, poor sperm morphology with subsequence sub or infertility

The upregulation of alternative splicing regulators, together with the loss of specific protein isoforms and their association with poor semen quality prompted us to test the link directly. For this cause, we focused on hnRNP H/F, for three reasons. Firstly, hnRNP H/F has just one orthologue in *Drosophila,* namely Glo making it easier to test. Secondly, there is precedence that Glo is involved in RNA regulation within germ cells.^22^ Finally, in human testis biopsies that underwent hyperthermia,^23^ and mice given a single heat event of testicular stress,^24^ hrnRNP H/F levels were shown to change; undergoing both a decrease, then increase in expression. As such, the expression level of hnRNP H/F can be physiologically regulated in the testis environment, making it a prime candidate to possibly cause poor semen quality.

**Figure 3:**
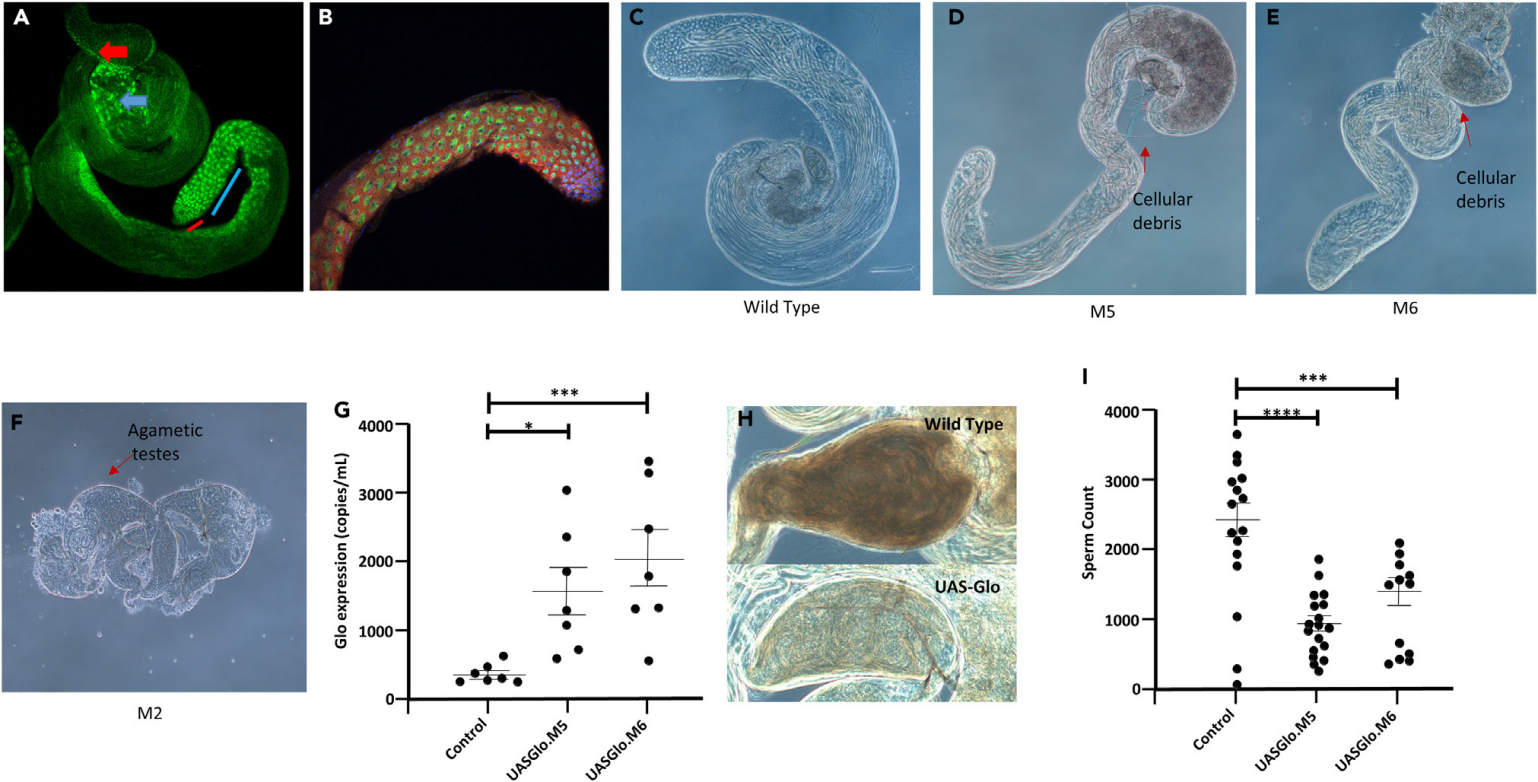
Upregulation of *Drosophila* Glo results in lower sperm production (A) A transgenic Glo-GFP fusion protein shows the endogenous expression pattern of Glo in the testis. Strong expression was observed in spermatogonia (red line) with expression decreasing in spermatocytes (blue line). High levels of expression were also observed in elongating spermatids (blue arrow) and epithelial cells of the seminal vesicle (red arrow). (B–E) Transgenic flies were produced with UAS-Gal4 system to drive transgene expression via the nos promoter (UAS-Gal4, nos-Gal4). Testis from either the (B) control (C) M5 (D) M6 or (E) M6 strain were isolated and imaged as shown. (F) Transgene expression levels in both M5 and M6 strains were quantified through digital PCR when expressed in developing eye tissue (ey-Gal4 promoter) as loss of germ cells confounded examination of transgene expression in testes. (G) Phase contrast image of a seminal vesicle from the control (above), or M5 strain (below). Less abundant spermatozoa are evident. (H) Phase contrast image the seminal vesicle taken from a Wild type (upper) and transgenic Glo strain (lower). (I) *ProtamineB-GFP* transgene was combined with *bam-Gal4* and either control, *UAS-Glo.M5 or UAS-Glo.M6* and the corresponding (non-overlapping) fluorescent spermatozoa were counted for sperm concentration. ∗ = *p* < 0.05, ∗∗ = *p* < 0.01, ∗∗∗ = *p* < 0.001, ∗∗∗∗ = *p* < 0.0001 (Student’s t test). Interquartile ranges are shown.

To determine the normal level of expression of Glo within the testis of *Drosophila*, we obtained a genetically modified strain where the endogenous protein has been tagged with GFP. As shown (Figure 3A), strong staining of Glo was present in spermatogonia (red line) and with reduced levels in spermatocytes (blue line). The germ cell localization suggested Glo was localized to chromatin, which is in accord with its role as an RNA binding protein. Notably, Glo was also found to be highly expressed in spermatids during individualization stage (blue arrow), suggesting Glo has a role in almost all stages of germ cell development, as well as in epithelial cells of the seminal vesicle (sperm storage organ, red arrow).

To understand the role of Glo and its involvement in spermatogenesis, we produced *Drosophila* mutants in which the protein was overexpressed, which is in accord with what we see in some of the human sperm samples. We utilized the bipartite UAS-Gal4 system to control gene expression where *Glo* was cloned behind a UAS promoter. Expression was driven in male germ cells from either the *nos* or *bam* promoters by mating to strains that carried *nos-Gal4* or *bam-Gal4* transgenes. The exogenous Gal4 transcription factor binds to the UAS promoter to drive *Glo* expression. We also selected the *bam* promoter as it drives expression in late spermatogonia and transgene expression often perdures through meiosis into maturing spermatids. The *nos* promoter drives expression in spermatogonia and we combined it with a *UAS-Gal4* feedforward expression driver to also facilitate post-meiotic gene expression.

**Figure 4:**
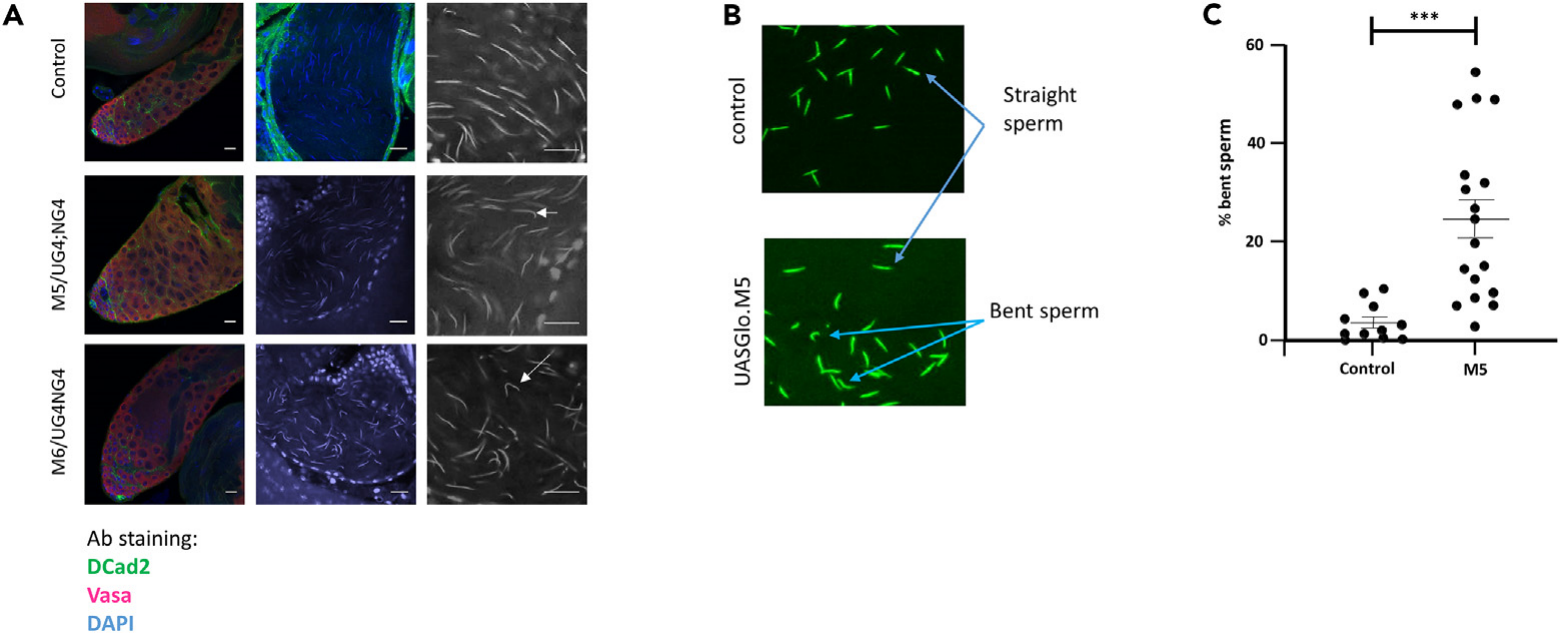
Upregulation of Glo causes sperm head morphology defects in *Drosophila* (A) Expression of UAS-Glo.M5 or UAS-Glo.M6 did not result in visible abnormalities in the apical region of the Drosophila testis (left panels) that contains spermatogonia and spermatocytes (germ cells—Vasa (red), somatic cells—DE-cadherin (green), nuclei—DAPI (blue)). Sperm nuclei stained with DAPI in seminal vesicles (middle panel). Higher magnification of sperm in seminal vesicles revealed abnormalities from germ cells with elevated Glo (right panel). (B) The *ProtamineB-GFP* transgene was combined with *bam-Gal4, +/− UAS-Glo.M5* and the corresponding spermatozoa were retrieved from the seminal vesicle. Image shows (non-overlapping) fluorescent spermatozoa from control (top) and M5 strain (bottom). (C) Graph of the percentage of bent spermatozoa present from spermatozoa taken form the seminal vesicle of either the control or the M5 strain. Scale bars, 15 μm. ∗∗∗ = *p* < 0.001, (Student’s t test). Interquartile ranges are shown.

Three transgenic strains were produced, designated *M2*, *M5*, and *M6* (Figures 3C–3E), with transgene insertion at different genomic locations, therefore, having the potential for expression at different levels due to differing chromatin environments. The *UAS-Gal4*, *nosGal4* combination was initially used to drive expression in germ cells. Compared to control (Figure 3B), Strain *M2* produced no spermatozoa, but rather cell death occurred in early germ cells to produce agametic testes (Figure 3E). In both *M5* and *M6* strains, inspection of testes via phase contrast microscopy revealed the presence of pre-meiotic and post-meiotic germ cells and an increase in cell debris near the basal testis (Figure 3C and 3D). Due to cell death, the determination of the level of gene expression from the three transgene insertions was problematic in the testis. To overcome this, we used *ey-Gal4* to drive transgene expression in developing eye tissue and then measured *Glo* levels in dissected heads with droplet digital PCR. The strain *M2* still resulted in lethality in combination with *ey-Gal4* and therefore was not measured. Strains *M5* and *M6* exhibited variable expression but overall 4.5- and 6.6-fold increases in expression from endogenous levels (Figure 3F). After upregulation of Glo was confirmed, we investigated its effect on sperm parameters. Examination of *UAS-Gal4*, *nosGal4/UAS-Glo* testes revealed that spermatozoa was present in 14/15 and 16/16 seminal vesicles from *M5* and *M6* strains, respectively, compared to 15/15 in control testes. However, when seminal vesicles were inspected, the transgenic flies had an obviously lower sperm density than controls. For example, Figure 3G depicts a higher density of sperm in the control compared to the Glo-upregulated seminal vesicle. To quantify this, we introduced a *ProtamineB-GFP* transgene into the *bam-Gal4*, *UAS-Glo.M5* background to allow us to accurately count sperm numbers in seminal vesicles. The seminal vesicles of *M5* (*n* = 18) and *M6* (*n* = 15) exhibited a 62% and 53% decrease, respectively, in the number of sperm when compared to controls (*n* = 15) (Figure 3I). These data demonstrate that upregulation of *Glo* leads to a significant loss in sperm production. In addition, the *ProtamineB-GFP* enabled the examination of sperm morphology. From this analysis, it was clear that the sperm heads of Glo-upregulated flies exhibited morphological abnormalities. Our initial examination of *UAS-Gal4*, *nosGal4/UAS-Glo* testes indicated no abnormalities in the apical region, which contain spermatogonia and spermatocytes, of M5 and M6. However, the nucleus of sperm cells present in the seminal vesicles exhibited morphological aberrations after upregulation of Glo (Figure 4). For example, the control group (Figure 3A, top) demonstrates the typical “needle-like” structure of *Drosophila* sperm nuclei. However, upregulation of Glo lead to abnormal sperm head, typically a bent form (Figure 3A, middle and lower). To conservatively quantify this, we counted non-overlapping sperm heads from control and *bam-Gal4*, *UAS-Glo.M5* seminal vesicles that also express ProtamineB-GFP (Figure 4B). While control sperm exhibited 3% morphological defects (149/4891, *n* = 15 seminal vesicles), upregulation of Glo led to 20% (851/4263, *n* = 18 seminal vesicles) of sperm heads with bent or hook shape (Figure 4C).

**Figure 5:**
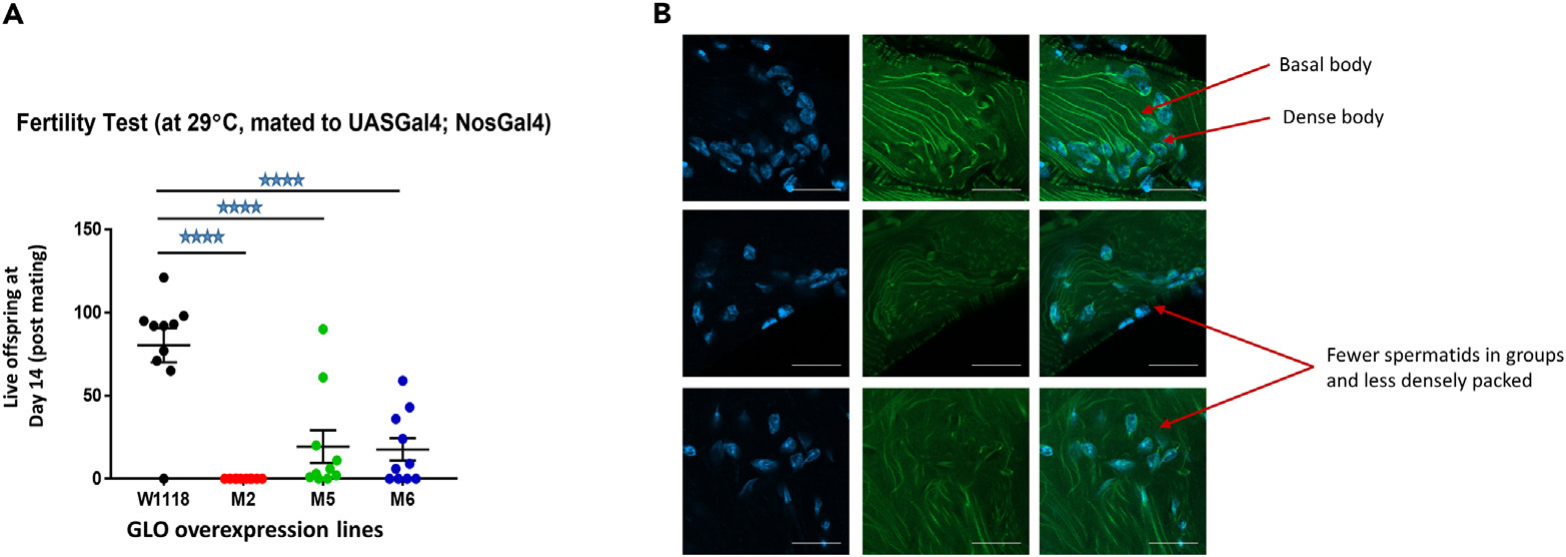
Upregulation of Glo causes male fertility defects and poor sperm morphology in *Drosophila* (A) Fertility of at least 10 individual males per genotype was examined after mating with females and the number of live offspring at Day 14 (post mating) were counted. (B) The male reproductive tract from *Drosophila* was obtained from control (top), M5 (middle) or M6 (bottom) that also carried a β-tubulinGFP fusion protein. The cells were stained with DAPI (left) or anti-GFP (middle, right). The basel body and dense body are indicated in the controls (top). Less densely packed spermatids are indicated in the M5 and M6 strains. ∗∗∗∗ = *p* < 0.0001(Student’s t test), Interquartile ranges are shown. Scale Bar, 20 μm.

To test the impact that a reduction in sperm number and an increased in abnormal forms would have on fertility, we next quantified the fertility of individual control and *UAS-Gal4*, *nosGal4/UAS-Glo Drosophila* strains. Fertility tests using 10 individual males showed strain *M2* was infertile. This was expected as this strain produced no spermatozoa. Fertility trials of strains *M5* and *M6* resulted in 24% and 22% of progeny production, respectively, compared to the level observed in control males (Figure 5A). Thus, it is clear that upregulation of the alternative splicing regulator *Glo* leads to dramatic changes in sperm number and morphology. This in turn leads to either major sub-fertility or infertility, which is consisted with the original human datasets for which this data were based.

### The nature of the spermatogenic defect

In order to understand how upregulation of Glo leads to poor sperm production, we investigated the spermatogenic pathway*. Drosophila* spermatid nuclei undergo a dramatic morphological change during maturation, going from a round nucleus, immediately post-meiosis, to a canoe shape nucleus, during late spermatid elongation, and then to a needle shape nucleus, after replacement of histones with protamines. Spermatids remain connected via ring canals until the very last stage of spermiogenesis. Therefore, bundles of 64 sister spermatids showing nuclei at the same stage of development and each connected via a basal body to an axoneme can be seen. Parallel axonemes can be observed trailing from clusters of similarly condensed nuclei. Nuclear shaping is also facilitated via a bundle of microtubules that wrap around one side of the nucleus, termed the dense body. To understand the basis by which overexpression of Glo causes poor sperm morphology in transgenic *Drosophila*, we introduced a *β-tubulin-GFP* transgene into *UAS-Gal4*, *nosGal4/UAS-Glo.M5 or UAS-Glo.M6* animals to examine the structure of axonemes, basal bodies, and dense bodies (Figure 5B).

Control flies demonstrated a prominent straight microtubule bundles in axonemes, and a cluster of nuclei undergoing synchronous condensation/shape change (Figure 5B, upper panel). However, transgenic strains showed less prominent dense bodies, with wavy bundles in the axonemes (Figure 5B, middle and lower panels) and dispersed nuclei with different stages of condensation in the same cluster. The dense bodies are analogous to the mammalian structure known as the manchette; a transient skirt-like structure that surrounds the head of elongating cells and helps shape them into morphologically normal sperm. In animal knockout models, each time a gene associated with manchette formation is deleted, the mice are either infertile or sub fertile, with sperm morphology defects. Examples include *KIF3A*,^25^ *MNS1*,^26^ *HOOK1*,^27^ *RIM-BP3*,^28^ MEIG1^29^ *SPEM1*,^30^ and katanin p80.^31^ Similarly, overexpression of *Glo* in the *Drosophila* led to poor dense body formation, tangled axonemes and poor-quality spermatozoa. Taking all together, the defects noted on the microtubules are presumably responsible for the lack of synchronous nuclear shaping, which consequently resulted in the bending of sperm heads observed in seminal vesicles.

## Discussion

The production of a limited amount of low-quality spermatozoa is attributed to abnormal processing of biochemical pathways and/or proteins occurring during spermatogenesis. However, understanding the mechanistic reasons behind this is an extremely difficult task due to the inaccessibility of human testicular tissue, which needs to be obtained in significant quantities from both fertile and infertile individuals. One strategy to overcome this, is to examine the composition of spermatozoa, specifically focusing on the variations between fertile and infertile individuals. The objective of this analysis is to gain valuable insights into the underlying factors that contribute to the development of these cells, thereby shedding light on the processes leading up to their formation. This approach has proven to be beneficial, as proteomic studies that look at single isolated cases such as asthenozoospermia^32^^,^^33^^,^^34^^,^^35^^,^^36^^,^^37^^,^^38^^,^^39^^,^^40^^,^^41^ or globozoospermic^32^ and azoospermia^42^ have shown involvement of essential proteins.

Our study, which consisted of oligoteratozoospermic patients shows 369 protein changes. A comparison of our data to that of similar work, which looked at protein changes in men with severe oligoasthenoteratozoospermia (OAT), shows RNA regulation pathway is indeed highly enrichment (1^st^ in our analysis and 2^nd^ in others). Furthermore, like our study, men with OAT show changes in Glo-orthologue hnrnpH/F.^43^^,^^44^ Furthermore, aberrant regulation of hnrnpH/F has been reported in asthenozoospermia patients^34^^,^^35^ and men undergoing hyperthermia,^45^ suggesting this protein is highly associated with poor human semen quality.

The idea that aberrant RNA regulation may lie at the heart of poor semen quality is intriguing, as spermatozoa are well known to be transcriptionally and translationally silent at the nuclear level. Thus, changes in RNA profiles would occur well upstream, typically weeks before sperm formation. Yet the paradigm appears to hold true. The double knockout mice for ADAD2, a double-stranded RNA binding protein expressed at the pachytene stages onwards, produced a lower amount of MIWI and MILI piRNAs whereas transposon-derived secondary piRNAS are increased.^7^ In what was described as an “unexpected” result, one main role for piRNA in *Drosophila* is the regulation of alternative slicing.^46^ Furthermore, mice made functionally redundant in alternative splicing regulators hnRNP-T^15^ RBM and DAZ^16^ also produce poor quality semen profiles. In all cases, the proteins are expressed well upstream of sperm formation. In most cases, animals still produce sperm, albeit at much lower concentrations. Further to this, functional deletion of testis specific kinase 3 (TSSK3)^47^ and FAM17a^48^ also lead to male infertility with the mice producing similar phenotype describe here. Although the exact mechanisms of action for both proteins are not understood, it is interesting to note that both are located in the nucleoplasm and nuclear bodies, the site at which alternative splicing occurs. Furthermore, both are expressed at the pachytene stage of development. In the case of FAM17a, the knockout showed no evidence of transcriptional changes, thereby ruling out changes in RNA abundance and likely pointing to the involvement of other RNA regulatory mechanisms.^48^

What intrigues us, is how some germ cells do not undergo apoptosis during spermatogenesis, but manages to “tolerate” these changes. However, the analysis is proving to be very complex. Unlike other Hnrnp proteins which display a varying range of affinities for different ribonucleotides sequences and ssDNA, early reports suggested that hnrnpH/F, only bind poly(rG) *in vitro.*^49^ In doing so, they regulate exon skipping of c-SRC, Mcl-1, Tcf3, and Bcl-xl.^50^^,^^51^^,^^52^^,^^53^ In the case of the latter, hnrnpH/F promotes a splicing event that results in the formation of the pro-apoptotic protein Bcl-Xs rather than the anti-apoptotic protein Bcl-Xl.^52^ RNA splicing factors have been reported to exhibit dynamic expression patterns during spermatogenesis in *Drosophila*, with alternate splicing events more common in spermatogonia than more differentiated cells,^54^ consistent with the expression pattern of Glorund. Beyond exon skipping, hnrnpH/F has been shown to be involved in RNA regulation through alternative polyadenylation and stability.^55^^,^^56^^,^^57^^,^^58^^,^^59^ Interestingly, testes show enrichment for transcripts with alternative polyadenylation (shorter 3′UTR regions) compared to other cell types (63), which allows for unique regulation. The classic example of this is protamine 1 in which the transcript is produced in early haploid cells, but protein production does not occur until 5 days thereafter.^60^ The manner in which translational repression occurs has to do with the length of the 3` (poly A) region; an area that hnrnpH/F binds to. Altering the length of the Poly A tail lifts the suppression of the transcript.^57^ Further to this, regions within the polyA sites hnrnpH/F bind to is canonical RG3 sequences (G3A2G3A2G3A2G3). These sequences are typically present within cell stress response genes, including those involved in DNA damage.^58^ As such, not only may hnrnpH/F promote alternative splicing and the apoptosis promoting products, but also appears to suppress the ability of the cell to respond to stress through alternative poly-adenylation sites. Whether this gives tolerance to cells to proceed through the spermatogenic cycle is unclear.

A third notion into the role of hnRNP H1 specifically has come from work that produced a germline-specific knockout.^17^ Loss of hnRNP H1 within the testis leads to azoospermia, with detectable sperm present in the seminiferous tubules at stages VII-VIII.^17^ This very different from our overexpression model, where sperm are still produced albeit at a much lower concentration and of poor quality which suggests the testis appear to tolerate overexpression of the gene rather than complete loss. Both enriched spermatocytes and spermatids deplete of hnRNP H1 showed a large number of mRNA alternative splicing changes (∼800 in total) as would be expected. However, unexpectedly, within the pachytene spermatocytes and round spermatids, respectively, 3283 or 2892 genes were upregulated compared to only 310 or 599 genes which were down regulated in the hnRNP H1knockout model.^17^ These data adds weight to the idea that hnRNP H1 plays several roles apart from alternative splicing and may be involved in gene repression during spermatogenesis.

Although we see changes in many Hnrnp proteins, the question remains of whether they are physiologically relevant. In human, when men are given scrotal hyperthermia for 6 weeks, the level of hnrnpF significantly decreases at week 2 and 6, respectively.^61^ Thus, it is plausible that the level of hnrnpF expression is temperature-sensitive. Furthermore, in humans with an associated varicocele, the closely related exon skipping protein hnrnpM is only found in fertile, but not infertile spermatozoa.^62^ However, different animal systems may respond uniquely. In mice, a single heat event (water bath scrotal immersion, 42°C, 15 min) showed 37 protein changes. Although the level of hnrnpF was unaffected, other closely related alternative splicing regulators hnrnpA2/B1, hnrnpE1, hnrnpX, and hnrnpK showed a significant change in expression.^63^ As such, there is clear evidence that testicular hyperthermia impacts the abundance of different alternative splicing regulators, which in turn is highly likely to impact sperm production.

In summary, we show that overexpression of several alternative splicing regulators is a hallmark of oligoteratozoospermic samples. Remarkably, the overexpression of just one of the regulators is enough to cause the exact condition with evidence of male sub and infertility. Increased testicular temperature may be a mechanism by which these regulators change in terms of protein expression, albeit different animal models are likely to respond in different ways.

### Limitations of the study

One limitation of these data are that they primarily focus on a specific subset of proteins and their potential role in male infertility. While the quantitative proteomic analysis and subsequent investigations provide valuable insights into potential mechanisms underlying subfertility or infertility, they may not capture the full complexity of the condition. Secondly, the study predominantly relies on animal models, specifically *Drosophila*, to explore the functional consequences of dysregulated proteins on reproductive pathology and fertility. While animal models can provide valuable insights, there may be differences in biological processes and regulatory mechanisms between species, limiting the direct translation of findings to human infertility.

## STAR★Methods

### Key resources table

**Table:** 

REAGENT or RESOURCE	SOURCE	IDENTIFIER
**Antibodies**		

anti-cathelicidin antimicrobial peptide (CAMP)	Proteintech	Cat# 12009-1-AP; RRID:AB_908736
anti-HSP4AL	Abcam	Cat# ab87241; RRID:AB_2119828
anti C1orf56 (MENT)	Sigma-Aldrich	Cat# HPA037430; RRID:AB_2675473
anti-beta Tubulin	Abcam	Cat#ab6046; RRID:AB_2210370
anti-Vasa	Santa Cruz Biotechnology	Cat# sc-26877; RRID:AB_793877
anti-DE-cadherin	Developmental Studies Hybridoma Bank	Cat# DCAD2; RRID:AB_528120
Goat Anti-Rabbit HRP	Merk	Cat# DC03L; RRID:AB_437852

**Chemicals, peptides, and recombinant proteins**		

Trizol reagent	Invitrogen	Cat#5596018
DNase I	Sigma	cat# D7291

**Critical commercial assays**		

SuperSignal™ West Atto Ultimate Sensitivity Substrate	Thermofischer	Cat#A38556
2-D quant kit	Cytivia	Cat#80648356
SensiFAST cDNA synthesis kit	Bioline	cat #BIO-65053

**Deposited data**		

Proteomics data	ProteomeXchange Consortium via the PRIDE	Project accession: PXD046594

**Experimental models: Organisms/strains**		

UAS-Gal4	Kyoto *Drosophila* Stock Center	108–492
GAL4:VP16-nanos.UTR	Kyoto *Drosophila* Stock Center	BL4937
Bam-Gal4:VP16	Kyoto *Drosophila* Stock Center	BL80579
ey-Gal4	Bloomington *Drosophila* Stock Center	BL5534
ProtB-GFP	Bloomington *Drosophila* Stock Center	BL58406
GloGFP	Vienna Drosophila Resource Center	v318719
β-tubulinGFP	L. Quinn	see this paper/WellGeneetics Inc
UAS-Glo	N/A	UAS-Glo

**Software and algorithms**		

PeakView™	Sciex	Version 2.1
Perseus	MaxQuant	version 2.5.5
R-script	C.Krisp	see this paper
MS data	PRIDE	PXD046594

### Resource availability

#### Lead contact

Further information and requests for resources and reagents should be directed to and will be fulfilled by the lead contact, Mark Baker (mark.baker@newcastle.edu.au).

#### Materials availability

With regard to the Drosophila strains used in this study: UAS-Gal4 (Kyoto *Drosophila* Stock Center 108–492) was combined with GAL4VP16-nanos.UTR (BL4937) to generate UAS-Gal4; nosGal4. Bam-Gal4 (Bam-Gal4:VP16, BL80579), ey-Gal4 (BL5534) and ProtB-GFP (BL58406) were obtained from the Bloomington *Drosophila* Stock Center. GloGFP (v318719) was obtained from the Vienna Drosophila Resource Center. β-tubulinGFP was obtained from L. Quinn and w^1118^ from WellGenetics, Inc. All remaining unique/stable reagents generated in this study are available from the lead contact with a completed Materials Transfer Agreement.

#### Data and code availability

• The mass spectrometry data have been deposited to and is publicly available atthe ProteomeXchange Consortium via the PRIDE partner repository with the project name: The role of HnrnpF/H as a driver of Oligoteratozoospermia” and Project accession: PXD046594.
• The R code used for protein isoform quantification is included as Figure S1.
• Any additional information required to reanalyse the data reported in this paper is available from the lead contact upon request.

### Experimental model and study participant details

#### Human subjects

A total of 21 male between 28 and 50 years old and of European descent were recruited for this study. Exclusion criteria included Men with a known genetic disorder or chromosomal abnormalities, Men with a past history of post-pubertal mumps, chemotherapy/radiotherapy, orchitis or orchidectomy of any cause, Men who have presented or been treated for orchitis, epididymitis or urethritis in the past year, Any form of smoking (tobacco, marijuana etc) within the past year (self report), Men with a past or current history of a relevant infectious disease (chlamydia, gonorrhea, genital herpes, hepatitis B, HIV/AIDS or syphilis), Men with a chronic medical condition, including: Cancer, Chronic Obstructive Pulmonary Disease, Crohns disease, cystic fibrosis, diabetes, epilepsy, heart disease, multiple sclerosis, or Parkinson’s disease. Men with a serious psychiatric condition including autism, attention deficit-hyperactivity disorder, bipolar disorder, major depressive disorder and schizophrenia that may prevent compliance with the protocol

#### Ethics approval and consent to participate

The work was approved by the University of Newcastle Human ethics committee (H-2013-0319) and carried out in accordance with The Code of Ethics of the World Medical Association (Declaration of Helsinki) for experiments involving humans. Each patient had signed a written consent document to participate.

#### Drosophila strains

All strains were cultured on molasses-based food at 25°C except for GAL4 crosses, which were raised at 29°C.

#### Generation of UAS-Glo transgenes

The Glo cDNA was amplified from an embryo cDNA library and directionally cloned using NotI and XbaI into pUAST. This vector was used to generate transgenic strains by BestGene, Inc. Three separate insertion strains designated UASGlo.M2, UASGlo.M5 and UASGlo.M6 were used in this study.

### Method details

#### Preparation of human spermatozoa

The study population was comprised donors (28–50 years old) who were free of any detectable organic disease. Institutional and State Government ethical approval was secured for the use of human semen samples in this research program. The semen samples were produced by masturbation after a 2–5 days abstinence. After liquefaction (37°C for 1 h), semen analysis was performed in a blinded fashion with regards to the study group and for the most part according to the WHO guidelines to obtain pH, sperm concentration and motility. For sperm morphology, two slides were prepared of each sample and evaluation was performed according to Krugers strict criteria.^64^ Sperm smears were first air dried onto glass slides and stained with methylene blue/eosin. At least 100 cells were examined per slide, with a final magnification of ×1000.

#### Sample lysis

Sperm pellets were resuspended in a lysis buffer consisting of 1% (w/v) C7BzO [3-(4-Heptyl) phenyl-3-hydroxypropyl) dimethylammoniopropanesulfonate], 7 M urea, 2 M thiourea, and 40 mM Tris (pH 10.4) at a final concentration of 20 × 10^6^/100 μL and incubated for 1 h (4°C) with constant rotation. Supernatant (18 000 × g, 15 min, 4°C) was recovered and total protein was quantified using a 2-D quant kit (G.E. Healthcare, Sydney, Australia) following manufacturer’s protocol.

#### Protein digestion

Lysate was taken and reduced using 10 mM DTT for 30 min at 30°C and then alkylated with 20 mM iodoacetamide for 30 min at 30°C in the dark. A total of 10 μg of protein was precipitated using methanol and chloroform.^65^. Samples were incubated overnight (37°C) with trypsin at a 1:50 (trypsin/protein) ratio. The sample was subsequently centrifuged (10,000 xg, 15 min) and the supernatant containing the tryptic peptides was acidified (0.1% Tri-fluoro acetic acid).

#### LC-MS and SWATH-MS

LC-MS analysis of digested sperm cell lysates from fertile (*n* = 12) and infertile men (*n* = 11) were performed on an Ekspert NanoLC 400 with cHiPLC system (SCIEX) coupled to a TripleTOF 6600 mass spectrometer (SCIEX). In RP LC-MS/MS mode, a 200 μm × 0.5 mm nano cHiPLC trap column and 15 cm × 200 μm nano cHiPLC columns (ChromXP C18-CL 3 μm 120 Å) was used. For data dependent MS/MS acquisition, 20 most intense m/z values exciding a threshold >250 cps with charge stages between 2+ and 4+ were selected for analysis from a full MS survey scan and excluded form analysis for 20 s to minimize redundant precursor sampling. Peptides were separated over 120 min 5–35% acetonitrile (ACN) gradients. In data independent acquisition a 100 variable window method over a range of 400–1250 m/z with window sizes based on precursor densities in the LC-MS/MS acquisition. Collision energies were calculated for 2+ precursors with m/z values of lowest m/z in window +10% of the window width. The data were acquired over an 80 min 5 - 35% ACN gradient.

#### Protein identification

Spectral libraries for SWATH-MS quantitation were generated with ProteinPilot software 5.0 using the Paragon algorithm (SCIEX) in the thorough ID mode including biological modifications and chemical modifications. MS/MS data were searched against the human reviewed SwissProt database (release February 2016, 20,198 entries) and the human SwissProt database including reviewed protein isoforms (release May 2016, 42,168 entries) with carbamidomethyl as a fixed modification for cysteine residues. An Unused Score cut-off was set to 0.05 and the FDR analysis was enabled.

#### Data analysis

The generated Paragon group file was imported into PeakView software 2.1 using the SWATH MicroApp 2.0 (release 25/08/2014) to generate a sample specific spectral library which was matched against SWATH-MS data. After retention time calibration with endogenous peptides, data were processed using following processing settings; 100 maximal peptides per protein, maximal 6 transitions per peptide, peptide confidence threshold of 99%, transition false discovery rate <1%, 5 min extraction window and fragment extraction tolerance of 75 ppm. Shared peptides were not allowed for the initial comparison of fertile and infertile samples but were allowed for the isoform specific statistical analysis. Transition, peptide and protein areas of processed data were exported. Protein areas were log2 transformed and normalized by subtracting median protein areas per sample (Table S2) and were further analyzed using Perseus software version 2.5.5 to perform principal component analysis, Student’s T-test analysis with permutation based multi-variant testing and hieratical clustering.

#### Protein isoform mapping

Peptide area information after SWATH data extraction with PeakView2.1 shared and non-shared peptides were used to map peptide centric information to its corresponding protein group. An in-house R script (Supplementary 1) was developed to assign peptide areas to unique proteins, protein groups specific for protein isoforms, shared among protein isoforms or shared among multiple proteins. The script utilized the peptide area export from the SWATH microapp processed data and the distinct protein summary file exported from ProteinPilot. The distinct peptide summary file contains protein group information for each peptide. A lookup function was implemented to match the peptide in the SWATH peptide area export file to the protein groups in the distinct peptide summary file and protein group areas were generated summing peptide areas form peptide with identical protein groups. Final composed list was imported into Perseus software version 2.5.5 to perform data normalization and Student’s T-test analysis.

#### Immunostaining and microscopy

Testes were dissected from adult flies in PBS. They were either visualized live under phase contrast microscopy using a Zeiss Axioskop 2 microscope and images captured with Zeiss Zen software or fixed in 4% formaldehyde diluted in PBT (PBS +0.2% Triton X-100 (Sigma)) for 20 min. Testes were then washed and blocked in PBT +5% normal horse serum for 45 min before being incubated overnight at 4C in primary antibodies (goat anti-Vasa, 1:100, Santa Cruz Biotechnology Cat# sc-26877, RRID:AB_793877), rat anti-DE-cadherin (DSHB, 1:100, Cat# DCAD2, RRID:AB_528120) diluted in PBT, washed in PBS and incubated in secondary antibodies diluted in PBS for 2 h at room temperature. After washing testes were mounted in ProLong Gold Antifade Reagent with DAPI (Invitrogen) and imaged using a Zeiss AiryScan LSM800 or LSM880 laser scanning confocal microscope.

#### *Drosophila* sperm quantification

Control GAL4 and UAS-Glo strains were combined with ProtB-GFP to label sperm heads and seminal vesicles were separated from live testes in PBS. Seminal vesicle contents were spread in a drop of Ringer’s buffer (20μL) on a microscope slide and imaged with a Zeiss Axioskop 2 and Zen software. Sperm numbers were counted using FIJI image analysis software. Non-overlapping GFP-labelled bent sperm heads were manually counted.

#### RNA and cDNA sample preparation

UASGlo.M5, UASGlo.M6 and w^1118^ (controls) were crossed to EyGal4 at 25°C for 2–3 days before transferring to 18°C. Eclosed adults were transferred to 29°C for 48 h before heads were dissected(∼×10) and placed directly into Trizol in a microtube. RNA was purified as described by Invitrogen Trizol reagent User’s guide. Briefly, the tissue in Trizol (1mL) was manually homogenized using a plastic pestle before incubating for 5 min at room temperature, chloroform (200μL) added, mixed, and incubated for another 5 min. Samples were centrifuged at 12000g for 15 min at 4°C. The aqueous layer containing the RNA was transferred into a fresh tube. Glycogen (∼10μg) was added and mixed well. Isopropanol (500μL) was added, mixed by inverting tube several times and incubated for 10min at 4°C. This was followed by centrifugation for 10 min at 12000 g at 4°C. The supernatant was removed, 75% ethanol (1mL) added to wash pellet and centrifuged for 5 min at 7500 g at 4°C. Supernatant was discarded, pellet briefly air dried and resuspended in water (30μL). RNA quality and concentration was measured using the Agilent TapeStation (RNA Screen Tape, cat#5067–5576) and Invitrogen Qubit 4 Fluorometer (Qubit RNA broad Range assay kit, cat#Q10211) respectively. RNA (250ng) was DNase I (Sigma, cat# D7291) treated, followed by cDNA synthesis using Bioline SensiFAST cDNA synthesis kit (cat #BIO-65053).

#### Droplet digital PCR (ddPCR) - Gene expression

*Drosophila* Glo (Dm02139296_g1) and reference gene Rpl32 (Dm02151827_g1) predesigned Taqman gene expression assays were purchased from Thermo Fisher Scientific. The QX200 ddPCR BioRad system was used for gene expression assays following the BioRad ddPCR protocol. ddPCR Supermix for probes (no dUTP) (12.5uL) was mixed with 20× gene expression assay (1.25μL), cDNA sample (1uL) and water (10.25μL) to a final volume of 25uL. Mixed thoroughly by vortexing and centrifuged briefly. 20uL reaction mix was converted into approximately 20,000 nm droplets using the QX200 Droplet Generator and transferred into a 96 well PCR plate for thermal cycling using the C1000 Touch Thermal Cycler deep well reaction module. After thermal cycling, droplets were read on a QX200 droplet reader and assigned as either positive or negative based on fluorescence amplitude. Absolute quantification of target gene (copies/uL) was analyzed via QuantaSoft software.

##### Immunoblotting

Immunoblotting was performed as describe elsewhere.^66^ Nitocellulose blots were stained with anti-CAMP (Proteintech Cat# 12009-1-AP, RRID:AB_908736), anti-HSP4AL (Abcam Cat# ab87241, RRID:AB_2119828) and anti MENT (Sigma-Aldrich Cat# HPA037430, RRID:AB_2675473).

### Quantification and statistical analysis

All data were analyzed using Persueus (Maxquant) or Excel. The results are presented as the mean SEM values. The differences between the groups were assessed with a Student’s t test and *P*-value of less than 0.05 was considered statistically significant. Specific information regarding tests can be found in the figure legends. All data was assumed to be normally distributed for statistical analysis.

## Supplemental information

Document S1. Figure S1 and Tables S1 and S3

Table S2. Normalised log transformed quantitative data, related to Figure 2
