# Supplementary material for: The role of HnrnpF/H as a driver of oligoteratozoospermia
Source: iScience. 2024 Jun 6;27(7):110198. doi: 10.1016/j.isci.2024.110198 (PMC11292545; doi:10.1016/j.isci.2024.110198)
Supplement: Document S1. Figure S1 and Tables S1 and S3 [file mmc1.pdf]

iScience, Volume *27*

Supplemental information

The role of HnrnpF/H

as a driver of oligoteratozoospermia

Jacob K. Netherton, Rachel A. Ogle, Benjamin R. Robinson, Mark Molloy, Christoph Krisp, Tony Velkov, Franca Casagranda, Nicole Dominado, Ana Izabel Silva Balbin Villaverde, Xu Dong Zhang, Gary R. Hime, and Mark A. Baker

Table S1. Semen parameters used for this study, related to Table 1.
A) Semen anaylsis of individiual infertile donors.
B) Semen anaylsis of individiual fertile donors.

Panel A

Infertile			
VOLUME	MOTILITY	COUNT (10^6/mL)	MORPHOLOGY (% Normal)
4.0	32	33.75	6
4.0	53	45.03	5
4.0	49	13	8
5.0	39	20.7	7
3.8	53	14.145	4
2.5	53	42.9	7
4.5	37	8.05	7
2.5	45	36.63	6
4.0	44	5.535	4
5.5	39	18.9	4
1.5	45	33.75	4

Panel B

Fertile			
VOLUME	MOTILITY	COUNT (10^6/mL)	MORPHOLOGY (% Normal)
5	66	128	29
8	77	72	25
3	69	227	39
2	81	145	50
3	61	224	35
5	63	340	24
3.5	83	190	47
0.5	53	164	30
1.5	88	186	47
4	75	138	32

Table S3. Gene ontology analysis, related to Figures 1 and 2.
A) Shows the Sub-cellular compartment.
B) Shows the Biological Process.
c) Shows the Molecular Function of changing proteins.

Panel A

Sub Cellular compartment	# Proteins	%	P-Value	Benjamaini-corrected
Mitcochondrion	75	20.3	2.60E-18	1.20E-16
Nucleosome core	17	4.6	1.10E-11	2.60E-10
Cytoplasm	161	43.5	4.90E-11	7.50E-10
Proteasome	10	2.7	6.40E-07	7.30E-06
Cilium	17	4.6	1.80E-04	1.60E-03
Mitochondrion inner membrane	18	4.9	2.30E-04	1.80E-03
Flagellum	10	2.7	8.50E-04	5.60E-03
Cytoskeleton	41	11.1	2.40E-03	1.40E-02
Intermediate filament	7	1.9	3.40E-03	1.60E-02
Dynein	5	1.4	3.60E-03	1.60E-02
CF(1)	3	0.8	5.00E-03	2.10E-02
Chromosome	20	5.4	1.60E-02	6.10E-02
Virion	5	1.4	1.70E-02	6.10E-02
Cytoplasmic vesicle	21	5.7	3.90E-02	1.30E-01
Keratin	7	1.9	8.40E-02	2.60E-01

Panel B

Bioloigical Process	# Proteins	%	P-Value	Benjamaini-corrected
RNA binding	82	4.6	1.20E-07	8.40E-06
structural constituent of chromatin	16	4.3	3.40E-10	1.00E-07
unfolded protein binding	17	4.6	2.80E-09	5.50E-07
protein domain specific binding	22	5.9	8.70E-09	1.30E-06
protein binding	284	76.8	2.20E-08	2.60E-06
chaperone binding	14	3.8	1.30E-07	1.30E-05
protein heterodimerization activity	23	6.2	2.40E-06	2.00E-04
structural molecule activity	16	4.3	3.90E-06	2.90E-04
structural constituent of ribosome	15	4.1	1.40E-05	9.30E-04
cadherin binding	19	5.1	3.20E-05	1.90E-03
protein binding involved in protein folding	8	2.2	4.30E-05	2.10E-03
identical protein binding	56	15.1	4.30E-05	2.10E-03
ATP binding	52	14.1	4.70E-05	2.10E-03
ADP binding	7	1.9	4.90E-05	2.10E-03
ubiquitin protein ligase binding	16	4.3	8.30E-04	3.30E-02
heat shock protein binding	7	1.9	1.00E-03	3.90E-02
magnesium ion binding	13	3.5	1.50E-03	5.30E-02
flavin adenine dinucleotide binding	7	1.9	1.70E-03	5.60E-02
serine-type carboxypeptidase activity	3	0.8	3.40E-03	1.00E-01
oxidoreductase activity	12	3.2	3.70E-03	1.10E-01
catalytic activity	9	2.4	4.50E-03	1.20E-01
pyruvate dehydrogenase (NAD+) activity	3	0.8	5.00E-03	1.20E-01
L-lactate dehydrogenase activity	3	0.8	5.00E-03	1.20E-01
proton-transporting ATP synthase activity, rotational mechanism	4	1.1	5.00E-03	1.20E-01
MHC class I protein binding	4	1.1	5.80E-03	1.30E-01
nuclear import signal receptor activity	4	1.1	5.80E-03	1.30E-01
peroxiredoxin activity	3	0.8	6.90E-03	1.50E-01
ligase activity	5	1.4	7.70E-03	1.60E-01

Panel C

Molecular function	# Proteins	%	P-Value	Benjamaini-corrected
Isomerase		4.6	1.20E-07	8.40E-06
Oxidoreductase	32	8.6	4.50E-06	1.60E-04
Chaperone	18	4.9	8.20E-06	1.90E-04
Ligase	14	3.8	2.50E-05	4.30E-04
Ribonucleoprotein	19	5.1	8.50E-05	1.20E-03
Aminoacyl-tRNA synthetase	7	1.9	1.50E-04	1.80E-03
Ribosomal protein	14	3.8	2.30E-04	2.30E-03
tRNA-binding	4	1.1	2.10E-02	1.50E-01
Elongation factor	4	1.1	2.10E-02	1.50E-01
Viral nucleoprotein	4	1.1	2.10E-02	1.50E-01
Antioxidant	3	0.8	3.40E-02	2.20E-01
Carboxypeptidase	4	1.1	4.60E-02	2.70E-01
Motor protein	7	1.9	7.10E-02	3.80E-01
Peroxidase	3	0.8	9.70E-02	4.90E-01

Figure S1. R-Script for identifying protein isoforms, related to Fig. 3.

```
# choose Swath library text file
df = read.table(file=file.choose(), header = T)
df1 = df[,c(1:2)]

library(dplyr)
IsoGroup = df1 %>% group_by(Sequence) %>%
  summarise(ProteinGroup = paste(Protein_Name, collapse=","))

write.table(IsoGroup, file = "IsoGroup.txt", sep = "\t", quote = F, row.names = F, col.names = F)

--
```
